# Hyperspectral imaging to characterize plant–plant communication in response to insect herbivory

**DOI:** 10.1186/s13007-018-0322-7

**Published:** 2018-07-06

**Authors:** Leandro do Prado Ribeiro, Adriana Lídia Santana Klock, João Américo Wordell Filho, Marco Aurélio Tramontin, Marília Almeida Trapp, Axel Mithöfer, Christian Nansen

**Affiliations:** 1 Research Center for Family Agriculture, Research and Rural, Extension Company of Santa Catarina, Chapecó, Santa Catarina Brazil; 2 Federal University of Fronteira Sul, Chapecó, Santa Catarina Brazil; 3 Department of Bioorganic Chemistry, Max Planck Institute for Chemical Ecology, Jena, Germany; 4 Department of Entomology and Nematology, University of California, UC Davis Briggs Hall, Room 367, Davis, CA 95616 USA; 5 State Key Laboratory Breeding Base for Zhejiang Sustainable Pest and Disease Control, Zhejiang Academy of Agricultural Sciences, 198 Shiqiao Road, Hangzhou, 310021 China

**Keywords:** Reflectance profiling, Phytocompounds, Plant stress signalling, Plant defences, Insect–plant interaction, Plant phenomics

## Abstract

**Background:**

In studies of plant stress signaling, a major challenge is the lack of non-invasive methods to detect physiological plant responses and to characterize plant–plant communication over time and space.

**Results:**

We acquired time series of phytocompound and hyperspectral imaging data from maize plants from the following treatments: (1) individual non-infested plants, (2) individual plants experimentally subjected to herbivory by green belly stink bug (no visible symptoms of insect herbivory), (3) one plant subjected to insect herbivory and one control plant in a separate pot but inside the same cage, and (4) one plant subjected to insect herbivory and one control plant together in the same pot. Individual phytocompounds (except indole-3acetic acid) or spectral bands were not reliable indicators of neither insect herbivory nor plant–plant communication. However, using a linear discrimination classification method based on combinations of either phytocompounds or spectral bands, we found clear evidence of maize plant responses.

**Conclusions:**

We have provided initial evidence of how hyperspectral imaging may be considered a powerful non-invasive method to increase our current understanding of both direct plant responses to biotic stressors but also to the multiple ways plant communities are able to communicate. We are unaware of any published studies, in which comprehensive phytocompound data have been shown to correlate with leaf reflectance. In addition, we are unaware of published studies, in which plant–plant communication was studied based on leaf reflectance.

## Background

Plant defenses to insect herbivory include a wide range of co-evolutionary adaptations, and they are intensively studied from evolutionary, ecological and crop management perspectives. The leaf tissue damage by herbivores elicit complex cascades of physiological responses in surrounding cells (locally responses) and expression of hundreds of genes, and they induce whole-plant hormonal responses [1–3]. Plant defenses to insect herbivory involves triggering of so-called “herbivore-associated molecular patterns” (HAMPs) [3, 4], which lead to synthesis and emission of volatile organic compounds (VOCs) [1–3]. The synthesis and emission of VOCs in response to herbivory may enable faster systemic plant defense responses than hormonal signaling via the vascular tissue [1]. Many VOCs are known to be involved in the regulation of plant responses to herbivory, including jasmonic acid (JA) [3, 5–7], salicylic acid (SA) [3, 8–10], and reactive oxygen species [11–13]. For instance, it was demonstrated experimentally that mechanically damaged sagebrush plants released a pulse of methyl jasmonate that induced direct resistance in wild tobacco [14]. Furthermore, wild tobacco plants with clipped sagebrush neighbors had increased levels of putative defensive oxidative enzymes (polyphenol oxidase) and showed reduced levels of leaf damage by grasshoppers and cutworms [15].

According to Maffei et al. [16], reactive oxygen species and intracellular calcium signatures belong to early events of plant defenses in response to biotic stressors, and they are responsible for most of the cascading biochemical reactions. The oxidative metabolism, particularly hydrogen peroxide synthesis, is related to a wide variety of reactions and signaling cascades, which are necessary for all aspects of plant growth and defense against biotic and abiotic stresses [11]. In addition, hydrogen peroxide is involved in: development of individual root hairs xylem differentiation and lignification, wall loosening and cross-linking, root/shoot coordination and stomatal control, and hypersensitivity reactions [13, 17]. In addition to aerial plant–plant communication, there is a growing body of research describing below-ground plant–plant communication through the roots [18–22].

To date, most studies of plant defenses to insect herbivory and plant–plant communication have been characterized and quantified by means of either headspace collection of volatiles in combination with gas chromatography/mass spectrometry analysis [23–25] or through invasive sampling of leaf tissue to quantify up/down regulations of phytocompounds [26–28]. In addition, experimental infestation of plants with arthropods is commonly used to confirm induction of plant defenses by a wide range of stressors and to confirm plant–plant communication [9, 29–35]. However, such arthropod bioassays have the potential to further alter plant traits and do not enabled non-invasive monitoring of plant response over time. Availability of a non-invasive technique to quantify plant responses to biotic stressors, such as, a herbivore, would greatly improve the ability to characterize plant responses to biotic stressors over time. Moreover, such techniques would enable more precise detection of when (based on time and intensity of stressor) significant physiological changes occur and therefore when invasive tissue sampling should be performed in order to optimize the likelihood of detecting physiological and molecular responses. A non-invasive method to detect and quantify plant–plant communication would be of considerable importance in ecological studies of insect herbivory and adaptive/evolutionary plant responses. Finally, reflectance based detection of spectral bands responding to plant–plant communication may be of considerable relevance to the application of remote sensing technologies in agricultural systems, as it may greatly enhance the ability to detect emerging insect infestation hotspots.

The research field of plant phenomics is largely driven by deployment of a range of molecular and imaging technologies [36, 37]. The interest in plant imaging as part of high throughput phenotyping and modern crop breeding, reflects the growing understanding of and appreciation for the many important and complex ways that growing plants respond to their surrounding environment and stressors, including physiological responses to herbivory by insects. For almost three decades, it is known that general leaf compounds, such as lipids, oils, protein, nitrogen, lignin, starch, cellulose, sugars, and chlorophyll can be quantified based on spectral information in specific portions of the solar incident spectrum [38, 39]. As part of applications of imaging technologies to studies of plant responses to stressors [37], a considerable body of research describes detection and characterization of chlorophyll content in growing plants [40–42] and more broadly detection of leaf reflectance responses to biotic plant stressors [43, 44]. Despite a large body of research into applications of imaging technologies to detect and characterize plant stress signals, there are only a limited number of studies, in which changes in reflectance features have been associated with phytocompounds other than leaf pigments, such as, chlorophyll [42, 45, 46]. An exception is a number of studies, in which the authors demonstrated association between specific reflectance features and leaf potassium content [47–49]. Use of leaf reflectance data to detect and characterize plant responses to abiotic and biotic stressors is based on the assumption that induced stress interferes with photosynthesis, chemical composition, and physical structure of the plant and affects the absorption of light energy and thus alters the reflectance spectrum of the plants [43].

**Fig. 1:**
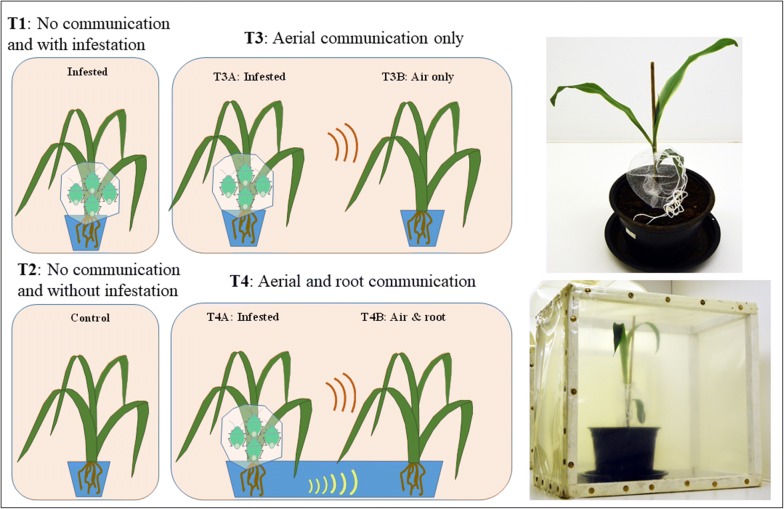
Illustration of the six plant treatments used in this study: (T1) a single maize plant in a cage infested with four stink bugs (2 males and 2 females), (T2) a single non-infested (control) maize plant in a cage, (T3) one infested plants (T3A) and one control (T3B) in the same cage but in separate pots, and (T4) one infested plants (T4A) and one control (T4B) in the in the same cage and in the same pot. T3 enabled plant–plant communication based on volatiles (air only) from the infested plant to the non-infested control plant. T4 enabled plant–plant communication based on volatiles and via roots (air and root) from the infested plant to the non-infested control plant. Photos of actual experimental units are included

In this study, the main hypothesis was that insect herbivory causes changes in leaf phytocompound levels, and these physiological defense responses are associated with detectable changes in phytocompound levels and in certain spectral bands of leaf reflectance profiles. As a secondary hypothesis, we predict that plant–plant communication (from plant with herbivory to an adjacent control plant without herbivory) will elicit both a change in phytocompound composition of leaves and also cause a corresponding change in leaf reflectance. To address these hypotheses, we acquired time series hyperspectral imaging data [before (baseline) and after 6, 12, 24, and 48 h of herbivory] from maize plants (*Zea mays* L.) inside cages subjected to the following four treatments (Fig. 1): (T1) individual non-infested plants (control). (T2) individual plants experimentally subjected to herbivory by green belly stink bug *Dichelops melacanthus* (Dallas) (Hemiptera: Pentatomidae). (T3) one plant subjected to insect herbivory (T3A) and one control plant (T3B) in separate pots but inside the same cage, and therefore the possibility of plant–plant communication via air space. (T4) one plant subjected to insect herbivory (T4A) and one control plant (T4B) together in the same pot, and therefore possibility of plant–plant communication via roots and air space. It is important to emphasize that this study was based on maize plants only being exposed to a low level of induced herbivory with no visible symptoms of insect herbivory. Using a separate set of maize plants under the same treatments, we obtained phytocompound data [jasmonic acid (JA), 12-oxo-phytodienoic acid (OPDA), auxins (in particular indole-3acetic acid (IAA)), salicylic acid (SA), hydrogen peroxide, carotenoids, and chlorophyll a and b] from all treatments and most time points. We are unaware of any published studies, in which comprehensive phytocompound data have been shown to correlate with leaf reflectance. In addition, we are unaware of published studies in which plant–plant communication was studied based on leaf reflectance. Thus, this study provides considerable novelty to the fields of insect–plant interactions and plant phenomics.

## Methods

### Test plants and insects

Potted (1 L) maize plants [*Zea mays* L. (Poaceae)] of hybrid Pioneer P2530 (non *Bacillus thuringiensis*) with four leaves [stage V3 [50]] were used in this study. Green belly stink bugs [*Dichelops melacanthus* (Dallas)] were obtained from a colony maintained in a climate-controlled room [temperature 25 ± 2 °C, relative humidity (RH) 60 ± 10% and a photoperiod of 14:10-h L:D] for at least 10 generations. Soybean [*Glycine max* (L.) Merrill (Fabaceae)] grains and plants were used as feeding and oviposition substrates, respectively. In Brazil, the green belly stink bug is a serious pest when feeding on young stems of maize plants [51]. Infestation of maize plants consisted of transferring two adult couples (four individuals) to a tulle fabric meshed bag (15 cm × 10 cm) [52], which was secured around the stem and lowest leaf of each maize plant (Fig. 1). Each experimental unit (either a single, treatments 1 or 2) or two, treatments 3 or 4 maize plant) were placed inside a wooden cage (30 × 25 × 25 cm), in which all six sides were covered with 100 µm plastic mesh. This choice of cages was to minimize the volatile exchange among experimental units, and at the same time enable some air to circulate to minimize humidity build-up and to ensure similar temperature conditions inside experimental units. All experimental units were kept in the same climate-controlled room (temperature 25 ± 2 °C, 60 ± 10% RH and 14 L: 10 D h photoperiod), with all experimental units exposed to the same lighting regime (illuminance not measured). All treatments were replicated five times for hyperspectral imaging and three times for phytocompound data collections. As cages remained closed at all times when inside the climate-controlled room and only opened for a few minutes to perform hyperspectral imaging, the risk of plant–plant communication was kept as low as possible.

### Hyperspectral imaging of maize leaves

We acquired time series hyperspectral imaging data: before (baseline) and after 6, 12, 24, and 48 h of herbivory. After baseline hyperspectral imaging, individual maize plants were randomly assigned to treatments. Hyperspectral imaging was performed in a room immediately adjacent to the climate-controlled room, where the experimental units were maintained. At each hyperspectral imaging event, a single cage at a time was transferred from the climate-controlled room to the hyperspectral imaging room, and hyperspectral imaging was completed within 1–2 min per plant. The order of maize plants and experimental units being imaged was randomized each time.

Hyperspectral imaging conditions and settings are similar to those described in a range of other hyperspectral imaging studies [49, 53–58]. All hyperspectral images were collected with artificial lighting from 15 W, 12 V light bulbs mounted in 2 angled rows, one on either side of the lens, with 3 bulbs in each row. A voltage stabilizer (Tripp-Lite, PR-7b, www.radioreference.com) powered the lighting. Ambient climate conditions were between 24–26 °C and 50–60% relative humidity. A piece of white Teflon was used for white calibration, and “relative reflectance” refers to proportional reflectance compared to that obtained from Teflon. Consequently, relative reflectance values ranged from 0 to 1. For each combination of maize plant and time point, we imaged an area corresponding to about 2 cm^2^ (equivalent to 64,000 pixels) on the second leaf after it had been placed in a horizontal position. Each hyperspectral image from a given combination of plant and time point was divided into two halves. A hyperspectral push broom camera (PIKA II, Resonon Inc., Bozeman, MT) was used. The objective lens had a 35 mm focal length (maximum aperture of F1.4) and was optimized for the visible and NIR spectra. The main specifications of the spectral camera are as follows: interface, Firewire (IEEE 1394b), output, digital (12 bit), 160 bands (spectral) by 640 pixels (spatial), angular field of view, 7 degrees, and spectral resolution of < 3 nm. We acquired reflectance in 240 spectral bands in the range from 380 to 1031 nm. However, to avoid spectral bands with low signal/noise ratio, we only included 230 spectral bands from 405 to 1012 nm, and they were spectrally 3X-binned (averaged) into 75 spectral bands with a spectral resolution of about 9 nm. We calculated average leaf reflectance in each of the spectral bands for all combinations of maize plant and time point and used these data for statistical analyses. With five time points (0, 6, 12, 24, and 48 h), six plant treatments (Fig. 1), five replications, and two hyperspectral images, a total of 300 average leaf reflectance profiles (observations) were included in the statistical analyses. In other words, the number of observations was 4 times the number of spectral bands (N = 75). This ratio between observations and spectral bands is very important for the robustness of the data classification based on average leaf reflectance. Moreover, model over-fitting due to the Hughes phenomenon or violation of the principle of parsimony [59] is a major concern when the number of explanatory variables is similar or exceeds the number of observations [44, 58, 60, 61].

### Phytocompound analyses

Phytocompound analyses were carried out using the second leaf of maize plants from all treatment groups (Fig. 1) before infestation (baseline) and after 12, 24, and 48 h. Three replicates were used for each combination of treatment and exposure time.

A relative quantification of four phyto-signaling compounds (IAA, JA, SA, and OPDA) was performed using a method adapted from Trapp et al. [28]. After collection leaves were immediately frozen in liquid nitrogen and storage at − 80 °C until the extraction.

The leaves were ground in a crucible, and 100 mg of ground tissue was transferred to 1.5 mL tubes and extracted twice with 1 mL of methanol (70%, v v^−1^). After the extraction, the solutions were dried in a SpeedVac and re-suspended in 100 µL of methanol containing 20 ng mL^−1^ of each internal standard (d5-IAA, d5-JA, d4-SA). These samples were analyzed by HPLC–MS/MS on an Agilent 1100 HPLC system (Agilent Technologies, Böblingen, Germany) connected to a LTQ Iontrap mass spectrometer (Thermo Scientific, Bremen, Germany) equipped with an Electrospray ionization source.

The chromatographic separation was performed in a Luna Phenyl-Hexyl column (150 × 4.6 mm, 5 μm; Phenomenex, Aschaffenburg, Germany), using gradient elution with formic acid (0.05%, v v^−1^) and methanol (with 0.05% of formic acid, v v^−1^) as mobile phases A and B, respectively. The elution profile was: 0–10 min, 42–55% B in A; 10–13 min, 55–100% B; 13–15 min 100% B; 15–15.1 min 100–42% B in A; and 15.1–20 min 42% B in A. The mobile phase flow rate was 1.1 mL min^−1^.

The ionization parameters were optimized for each phyto-signaling compound separately. Except IAA and d5-IAA (which were analyzed in positive ionization mode), all the others phyto-signaling compounds were analyzed in negative mode and monitored by the following SRM channels: IAA (176 > 130); d5-IAA (181 > 135); JA (209 > 59); d5-JA (214 > 62); OPDA (291 > 165); SA (137 > 93); d4-SA (141 > 97). Precursors and fragment ions were selected with an isolation window of 2 days.

Photosynthetic pigments (chlorophyll a and b, carotenoid) were measured based on fresh material from second leaves of maize plants (same as area being imaged with hyperspectral camera). Leaves of maize (fresh weight, 0.1 g) were frozen in liquid nitrogen and ground. Subsequently, ground leaf samples were transferred to a glass vial containing 5 mL of aqueous acetone solution (80%, v v^−1^) for 24 h, filtered and the absorbance was measured in an Analytik Jena spectrophotometer (Specord 200 Plus, Analytic Jena AG, Jena, Germany) at 663.6, 646.8 and 470 nm. Carotenoid and chlorophyll concentrations were calculated from the absorbance of extract at using the adapted formula by Lichtenthaler HK [62] as below:

$$ \begin{aligned} {\text{chlorophyll}}\;{\text{a}}\; ( {\text{mg}}\;{\text{g}}^{ - 1} )& = ( 1 2. 2 5\times {\text{A663,}}\; 6- 2. 6 9\times {\text{A646,}}\, 8 )\times 5 0 / 1 0 0 0\\ {\text{chlorophyll}}\;{\text{b}}\; ( {\text{mg}}\;{\text{g}}^{ - 1} )& = ( 2 1. 5 0\times {\text{A646}} . 8- 5. 1\times {\text{A663}} . 6 )\times 5 0 / 1 0 0 0\\ {\text{carotenoid}}\; ( {\text{mg}}\;{\text{g}}^{ - 1} )& = ( ( 1 0 0 0\times {\text{A470)}} - ( 1 ,\, 8 2\times {\text{Chl}}\;{\text{a)}}{-} ( 8 5 ,\, 0 2\times {\text{Chl b))/198)}} \times 5 0 / 1 0 0 0\\ \end{aligned} $$

To quantify hydrogen peroxide content, fresh tissue was collected from second leaf on maize plants (about 50 mg), and each sample was ground to a fine powder in liquid nitrogen and were homogenized in ice bath with 5 mL of trichloroacetic acid (0.1%, w v^−1^). The homogenate was centrifuged at 20,000 g for 30 min at 4 °C. The supernatant (0.5 mL) was added to 0.5 mL 10 mM potassium phosphate buffer (pH 7.0) and 1 mL of a 1 M L^−1^ potassium iodide solution. The absorbance of supernatant was checked at 390 nm [63]. The hydrogen peroxide content was calculated based on a standard curve prepared with hydrogen peroxide solutions (0–500 µM L^−1^) obtained by dilution of hydrogen peroxide (30%, w w^−1^).

### Experimental design and data analysis

Data processing and analyses were conducted in PC-SAS 9.4 (SAS Institute, NC). Initially, we conducted an analyses of variance (proc anova option = repeated and option = tukey) for repeated measures. An initial analyses of variance for repeated measures showed a highly significant effect of hours of insect herbivory (P < 0.001), and also a highly significant interaction between hours of insect herbivory and plant treatment (P = 0.001). The key focus of this study was to examine possible differences in maize plant responses to treatments, so effect of hours of insect herbivory was eliminated by rank-transformation of phytocompound data [ranked within each time point (0, 12, 24, and 48 h)] before further analysis. Using baseline data only (prior to insect herbivory), we conducted analysis of variance (proc anova) to examine the effect of the experimental design by comparing phytocompound levels and leaf reflectance from maize plants: (1) alone, (2) two plants in separate pots but in the same cage (T3A and T3B), and (3) two plants in the same pot (T4A and T4B). To analyze responses to insect herbivory and plant–plant communication, the maize plants were assigned to one of four groups:

- Control: baseline data from T1 and T2 plants, and all additional data acquired from T2 plants,
- Herbivory: all data acquired from T1, T3A, and T4A plants after onset of herbivory.
- Air only: all data acquired from T3B plants after onset of herbivory.
- Air and root: all data acquired from T4B plants after onset of herbivory.

We conducted analyses of variance (proc anova option = repeated and option = tukey) for repeated measures (with either rank-transformed phytocompound data or selected spectral bands) to compare average maize plant responses by these four groups.

The main data analysis was conducted in two separate parts, and, in separate analyses, we used the same experimental design in studies of phytocompound data and leaf reflectance data. Firstly, the purpose was to characterize maize plant responses to insect herbivory; secondly, the purpose was to characterize plant–plant communication. In order to characterize maize plant responses to insect herbivory, we examined data from the Control and Herbivory groups described above. In other words, maize plants that were either alone and not infested or plants that were directly infested, and a discriminant classification model (proc discrim) [64] was developed. Initially, stepwise linear discriminant analysis (proc stepwise) was used to only select the explanatory variables (phytocompound data or spectral bands) with significant contribution to each of the discriminant classification models. The selected subset of explanatory variables was used to generate the discriminant classification models, and their accuracies were quantified on the basis of 80% of the data being randomly selected as training data set and the remaining 20% of the data used for independent validation. This validation procedure was repeated 10 times to calculate average classification accuracies.

The second part of the main data analysis consisted of characterizing plant–plant communication, and this was conducted based on plants assigned to the “Air only” and “Air and root” groups described above. Moreover, the discriminant classification models (based on phytocompound data or leaf reflectance data from Control and Herbivory groups of plants) were used to interpret data from T3B and T4B plants. Under the assumption of plant–plant communication, T3B and T4B plants were predicted to be classified as subjected to herbivory (and not control).

## Results

### Phytocompound responses to insect herbivory

**Fig. 2:**
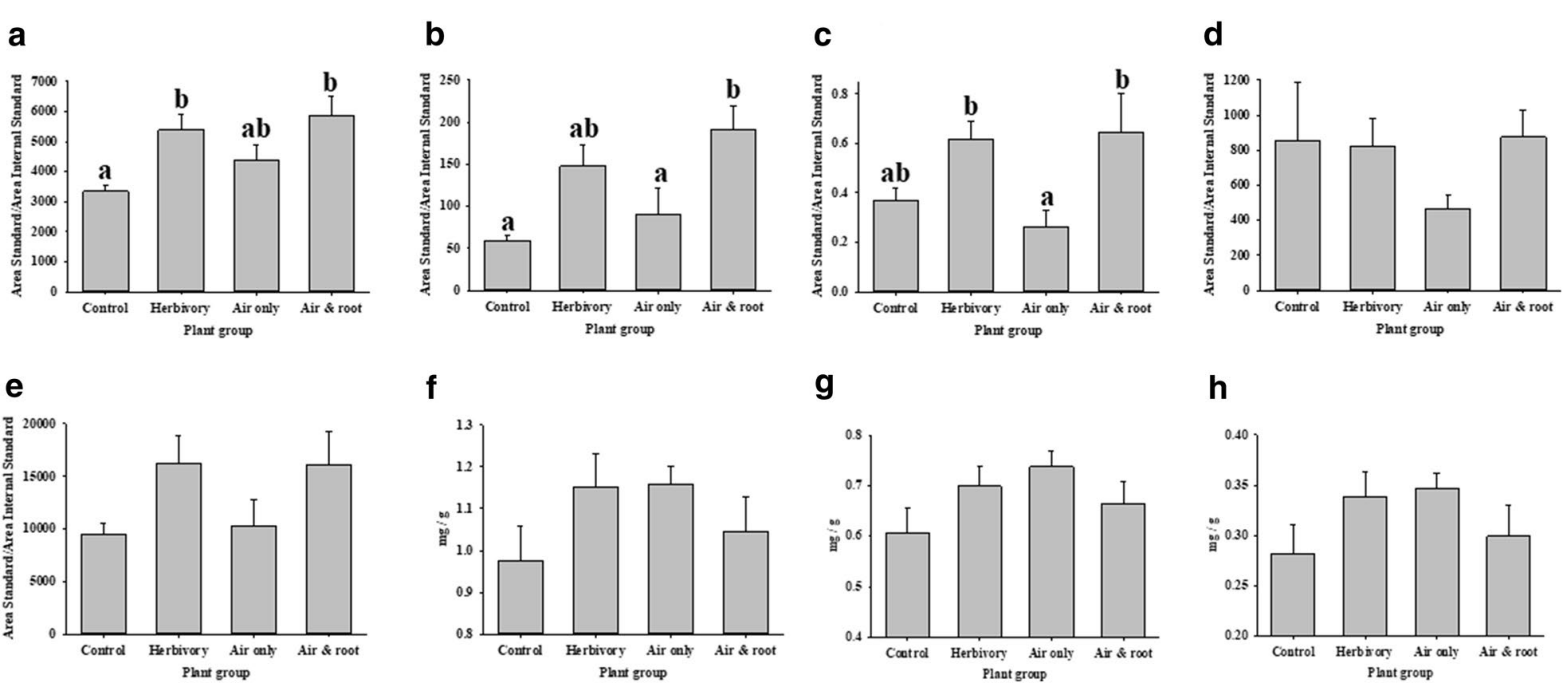
Average (error bars = standard error) phytocompound levels after maize plants had been divided into four groups (See Fig. 1 for treatment descriptions): “Control”: baseline data from T1 and T2 plants, and all additional data acquired from T2 plants, “Herbivory”: all data acquired from T1, T3A, and T4A plants after onset of herbivory, “Air only”: all data acquired from T3B plants after onset of herbivory, and “Air and root”: all data acquired from T4B plants after onset of herbivory. Different letters denote significant difference at the 0.05 level. Phytocompounds: [auxins (in particular indole-3acetic acid (IAA)), hydrogen peroxide (H_2_O_2_), jasmonic acid (JA), 12-oxo-phytodienoic acid (OPDA), salicylic acid (SA), chlorophyll a (Chl_a) and b (Chl_b), and carotenoids (Carot)]. **a** IAA, **b** JA, **c** H_2_O_2_, **d** SA, **e** OPDA, **f** chlorophyll a, **g** chlorophyll b, **h** carotenoids

At baseline, none of the eight phytocompounds varied significantly among the three groups of plants (P > 0.05). Thus, the experimental design (presence/absence of a second maize plant in the same cage) did not appear to significantly affect the phytocompound levels. Figure 2 shows average phytocompound levels after 12–48 h, after maize plants had been divided into four groups. Based on tukey comparisons, three phytocompounds (IAA, JA, and hydrogen peroxide) varied significantly among the four groups (P < 0.05), but only IAA showed a significant difference (up-regulation) in response to insect herbivory (Control vs. Herbivory).

Based on stepwise forward selection, we determined that IAA, hydrogen peroxide and chlorophyll a and b contributed significantly to the discriminant classification of Control and Herbivory plants. The validation of the discriminant classification model showed that the four phytocompounds could be used to classify Control and Herbivory plants with 80.7% accuracy (Control = 88.4% and Herbivory = 72.9%). Thus, although only IAA showed a significant up-regulation in response to insect herbivory (Fig. 2), a combination of phytocompounds provided strong indication of a maize plant response to insect herbivory. In following, the discriminant classification model was used to interpret phytocompound from T3B and T4B plants. We found that 67% of T3B plants were classified as Herbivory plants, which suggested that their phytocompound composition was more similar to that of plants subjected to insect herbivory than to control plants. We also found that 89% of T4B plants were classified as Herbivory plants. Thus, we demonstrated plant–plant communication, and this was particularly evident when root communication was possible.

### Reflectance detection of insect herbivory and plant–plant communication

**Fig. 3:**
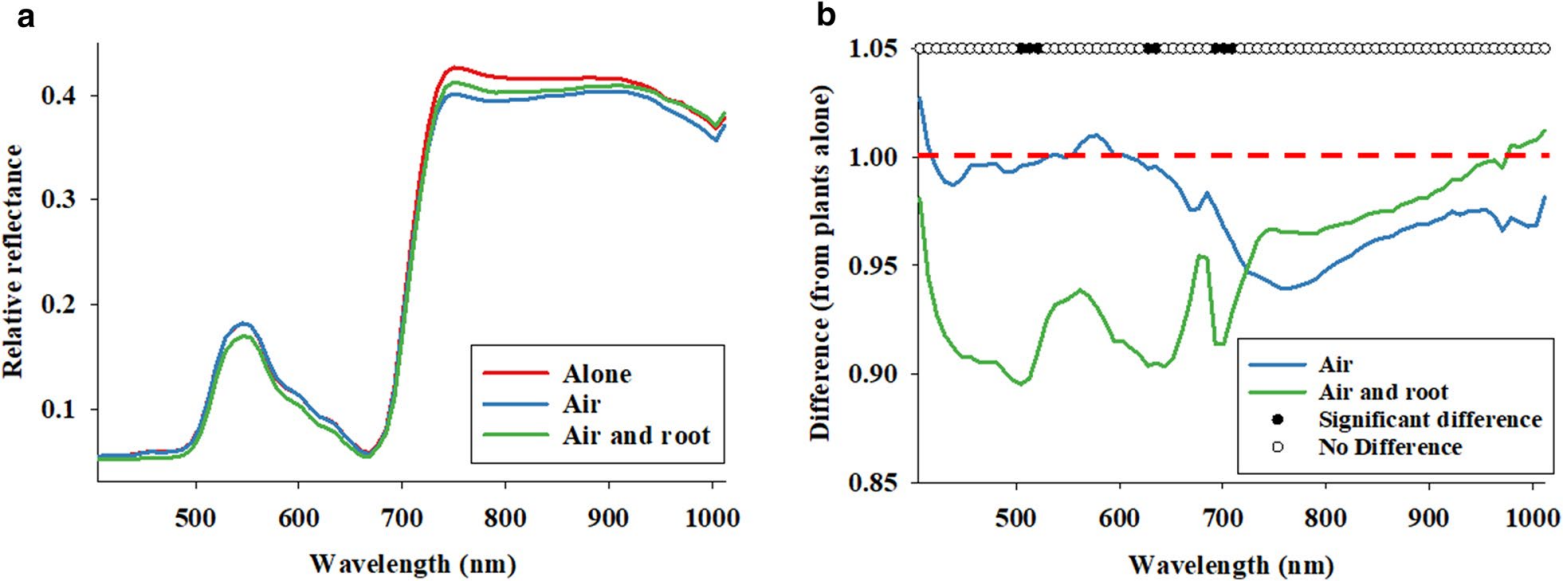
Average reflectance profiles **a** acquired from maize plants prior to insect herbivory (baseline) and divided into three groups: single plant per cage (alone), two plants in different pots but in the same cage (air), and two maize plants planted in the same pot (air and root). Average reflectance profiles **b** from T2, T3B, and T4B maize plants after 3–48 h of insect herbivory were divided with average reflectance profiles acquired from control plants (T1). See Fig. 1 for treatment descriptions. Significant F-values at the 0.05-level from statistical comparisons are denoted as dots

Figure 3a shows average baseline reflectance profiles of maize plants divided into three experimental groups. Average reflectance curves were also presented as their difference from that of Control plants (Fig. 3b). As the curves were generally < 1, it is seen that presence of a second plant inside cages generally caused a decrease in average leaf reflectance, especially in T4B plants in the spectral range from 405 to 700 nm. As indicated by black dots in Fig. 3b, eight spectral bands (near 500, 630, and 700 nm) showed significant difference in response to the experimental design.

**Fig. 4:**
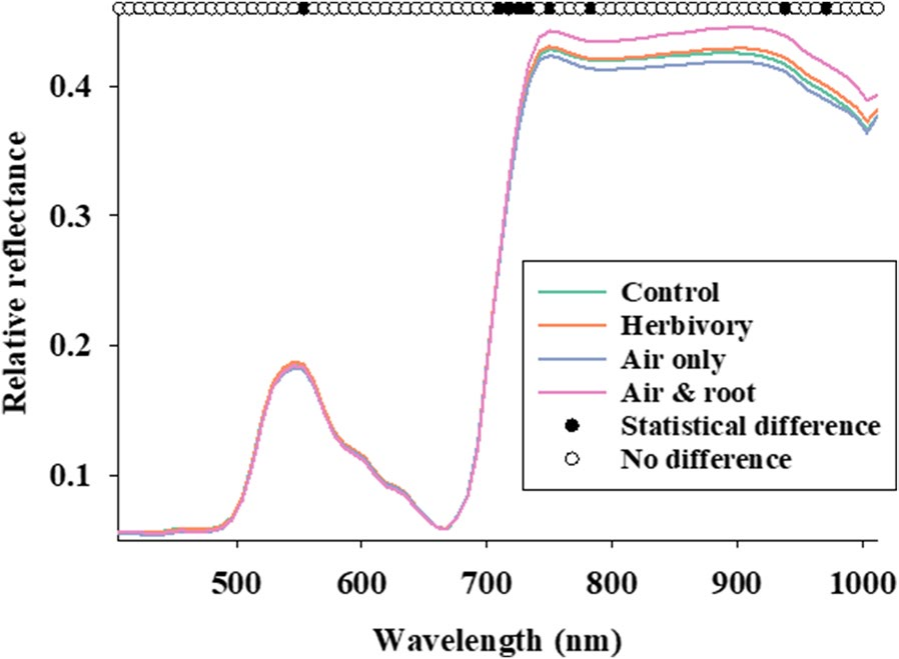
Average reflectance profiles from maize plants divided into four groups, “Control”, “Herbivory”, “Air only”, and “Air and root” (see Fig. 2 for description). Stepwise linear discriminant analysis was conducted to selected spectral bands (black dots), which contributed to the discriminant classification model of Control and Herbivory plants

Figure 4 shows the average leaf reflectance profiles of maize plants, when divided into four treatment groups. It is important to highlight that hyperspectral imaging data were acquired under controlled experimental conditions and that only a low and brief level of herbivory-induced stress level was imposed. Using stepwise forward selection, we found that 10 spectral bands (black dots in Fig. 4) contributed significantly to the discriminant classification model of Control and Herbivory plants. Based on analysis of variance for repeated measures, none of the 10 selected spectral bands showed significant difference in response to plant treatments (not shown). In other words, none of the spectral bands could be used individually as indicators of insect herbivory. However, we used the 10 spectral bands as explanatory variables in a discriminant classification model to classify Control and Herbivory plants, and this classification was associated with an overall accuracy of 79.3% (Control = 79.4% and Herbivory = 79.2%). Thus, despite only subtle between-class variation in average leaf reflectance data, it was possible to identify a reliable leaf reflectance response to low level of insect herbivory. In the second part of the hyperspectral imaging data analysis, the discriminant classification model (derived from plants assigned to Control and Herbivory) was used to interpret leaf reflectance data acquired from T3B and T4B plants. Interestingly, 76% (T3B plants) and 77% (T4B plants) were classified as subjected to insect herbivory. This result strongly suggested that plant–plant communication from infested plants (T3A and T4B) to T3B and T4B plants triggered a detectable leaf reflectance response, so that the average reflectance profiles of T3B and T4B plants were more similar to those of Herbivory plants.

## Discussion

This study was conducted to determine whether phytocompound data and hyperspectral imaging data could be used to detect maize plant response to both direct insect herbivory and also to plant–plant communication from an infested to a non-infested plant. We demonstrated that individual phytocompounds (except IAA) as well as reflectance in individual spectral bands were not reliable indicators of neither insect herbivory nor–plant communication. However, using a linear disciminination classification method based on combinations of either phytocompounds or spectral bands, we found clear evidence of maize plant responses. The potential of using changes in average reflectance data as indicator biotic stress is of particular interest to a broader community of researchers studying plant–insect interactions.

In a large body of literature, arthropods have been used as indicators of plant responses to a range of stressors and to plant–plant communication [9, 29–35]. As part of elucidating the mechanisms responsible for such bio-responses, it is well-established that herbivore-induced stress elicits plant physiological responses, which lead to changes in phytocompound levels (including OPDA, JA, IAA, and hydrogen peroxide) [1, 2, 65–67], and this was partially confirmed in this study. It is also well-described (and corroborated in this study) that herbivore-induced stress in one plant can elicit a phytocompound response in adjacent control plants, either induced by VOCs [1, 2, 14, 15, 68–70] and/or through root-based signaling [18–22]. Finally, there is ample evidence of how biotic stress by herbivores induces detectable changes in leaf reflectance [43, 44]. However, this is the first report describing herbivore-induced stress in plants subjected to insect herbivory causing a detectable and statistically significant change in leaf reflectance in an adjacent control plant. Thus, we are providing the first report of plant–plant communication of biotic stress being detected and quantified through the means of non-invasive method, namely analyses of leaf reflectance.

Plants have developed various strategies to defend themselves against herbivores and pathogens either alone or in “community” tactics [1]. Although some of these strategies are constitutive, most are inducible in response to stressors, so that plants enter a “primed state” [71, 72]. In general, induced stress interferes with photosynthesis, chemical composition, and physical structure of the plant and affects the absorption of light energy and thus alters the reflectance spectrum of the plants under stress [43]. In plants, immediate responses to herbivory are typically followed by a rapid and transient generation of reactive oxygen species in response to damage in the plant tissue [11–13, 73]. In a concentration-dependent manner, the hydrogen peroxide is a reactive oxygen species responsible by important functions in plant development and metabolism such as homeostasis, acclimatization, and plant defense processes [74]. In addition, hydrogen peroxide acts as a signaling molecule in plants to a variety of others signaling molecules and plant hormones [14, 75, 76], since it can induce the synthesis or activation of transcription factors that are associated with the induction of several enzymes of the antioxidant system [77, 78].

Literature reviews of hyperspectral vegetation indices have identified spectral bands in the 900–940 nm region to correlate well with wet biomass and leaf area index across a series of crops [79, 80]. It is also been determined that spectral bands near 948, 975 and 1004 nm are meaningful when developing spectral band indices to detect iron deficiency in crops [81]. In addition, the relationship reflectance at 970 nm/900 nm was proposed as a meaningful water index of crops [82]. However, the vast majority of hyperspectral indices are based on spectral bands from 450 to 800 nm [79, 81–85]. In the current study, eight spectral bands (near 500, 630, and 700 nm) showed significant difference in response to the experimental design (Fig. 3b). This result implies that some level of plant–plant communication was already occurring prior to the onset of insect herbivory. We also demonstrated that a combination of 10 spectral bands (see black dots in Fig. 4), especially between 700 and 750 nm, could be used to classify maize plants with/without insect herbivory. Most studies of reflectance responses to insect-induced stress have shown that herbivory causes an increase in reflectance [44]. Sensitivity analyses across wide spectral ranges (between 400 and 2500 nm) have shown that increased reflectance near 700 nm wavelength is a consistent indication of stress [86, 87]. Moreover, an increase in reflectance near 700 nm appears to be linked to reduced absorption due to lower chlorophyll concentration [42, 46, 88]. However especially regarding piercing-sucking herbivores, there are important exceptions suggesting that feeding behavior may be important. As an example, greenbug [*Schizaphis graminum* (Rondani) (Hemiptera: Aphididae)] infestations in wheat [*Triticum aestivum* L. (Poaceae)] caused a decrease in reflectance in the UV-light portion of the spectrum [89]. In addition, herbivory by bird cherry-oat aphids [*Rhopalosiphum padi* (L.) (Hemiptera: Aphididae)] also caused a decrease in leaf reflectance [90]. In a study of Russian wheat aphids [*Diuraphis noxia* (Mordvilko) (Hemiptera: Aphididae)] and greenbugs (*S. graminum*), both sucking herbivorous insects, it was determined that the strongest reflectance responses were detected in spectral bands between 625–635 and 680–695 nm [89]. The results presented in this study underscore the importance of disentangling reflectance responses associated with inherent and more general plant–plant communication from those being potentially providing specific information about signaling of biotic stress imposed by herbivorous insects. As this is the first study of plant–plant communication and leaf reflectance data, we do not have comparative data.

An important detail in this study was the structure and handling of cages used to hold the maize plants. That is, we decided to keep all cages in the same room, as it was considered critical that they were all under the same abiotic conditions (i.e. light, temperature and relative humidity). In addition due to concerns about mold and build-up of humidity (especially when two plants were in a cage compared to cages with one plant), it was considered necessary to allow some air movement in and out of cages. Also, use of hermetically closed cages could also affect the feeding behavior and survival of the stink bugs. However at the same time, we obviously had concerns about possible volatile-based communication among plants in different cages. It is possible that more significant plant responses would have been detected, if we had been able to control and maintain air flow inside each of the cages, but that was not possible due to logistical limitations.

In conclusion, we demonstrated plant–plant communication from an infested to a non-infested plant when based on phytocompound data as well as on average leaf reflectance data from a selected combination of spectral bands. Thus, we have provided initial evidence of how remote sensing may be considered a powerful non-invasive method to increase our current understanding of both direct plant responses to biotic stressors but also to the multiple ways plant communities are able to communicate. Due to plant–plant communication, our results suggest that in laboratory studies thorough caging and/or separation of replicated plants, and of plants subjected to different treatments, is of major importance when conducting experimental laboratory studies. We are aware of how growth chamber space at universities is often shared by students and faculty from multiple groups. In addition, multiple plants may be grown in flats or trays to save space. The results presented here suggest that interference via plant–plant communication may adversely affect the quality and/or consistency of the data being collected, unless individual plants are effectively “protected” from plant–plant communication. In an applied agricultural context, the results from this study are encouraging as they suggest that “hotspot” detection of emerging insect infestations in agricultural crops may be more likely if based on analyses of reflectance values in spectral bands. That is, if a small cluster of crop plants are infested by insects and emit a stress signal to adjacent plants, which in turn also respond to the stressor, then it may be easier to spatially localize the emerging infestation hotspot in crop fields. Thus, confirmation of leaf reflectance based detection of plant–plant communication is highly relevant to development of accurate and reliable methods in precision agriculture to determine when and where crop plants are under stress.
